# Associations between socioeconomic status, dietary habits and health-related quality of life among children in rural riverside communities: the mediation role of food insecurity

**DOI:** 10.1007/s11136-025-04137-0

**Published:** 2026-01-09

**Authors:** Luziane de Lima Pereira, Fernando José Herkrath, Jordana Herzog Siqueira, Maria do Carmo Leal, Fabíola Macedo de Abreu, Amanda Forster Lopes, Mario Vianna Vettore

**Affiliations:** 1 Fundação Oswaldo Cruz, Instituto Leônidas & Maria Deane, Manaus, Amazonas Brazil; 2 Programa de Pós-graduação em Saúde Coletiva, Universidade do Estado do Amazonas, Manaus, Amazonas Brazil; 3 Fundação Oswaldo Cruz, Escola Nacional de Saúde Pública, Rio de Janeiro, Rio de Janeiro Brazil; 4 Departamento de Alimentos e Nutrição, Universidade Federal de Santa Maria, Santa Maria, Rio Grande do Sul Brazil; 5 Department of Dentistry and Oral Health, Aarhus University, Vennelyst Boulevard 9, Aarhus C, 8000 Aarhus, Denmark

**Keywords:** Children, Quality of life, Food insecurity, Food quality, Rural population

## Abstract

**Purpose:**

This study investigated the relationships between socioeconomic status (SES), housing conditions, BMI *z*-score, food availability and consumption, food insecurity, and health-related quality of life (HRQoL) in schoolchildren aged 5 to 10 years living in rural riverine communities.

**Methods:**

This school-based cross-sectional study included 128 parent–child dyads living in rural riverside in the city of Coari, Amazonas, Brazil. SES, housing conditions, household food availability, consumption of ultra-processed foods and food insecurity data were gathered from children’s parents. Children’s nutritional status (BMI *z*-scores), diet quality and HRQoL [Kiddo-KINDL] were also assessed. Direct and indirect relationships between variables were examined through structural equation modelling.

**Results:**

Food insecurity was directly linked to poorer HRQoL. Worse socioeconomic status, lower availability of food at household, and poorer child’s diet quality were directly linked to food insecurity. Greater household food availability, higher children’s BMI *z*-score, and worse diet quality were directly linked to higher socioeconomic status. Availability of food at home was directly associated with higher consumption of ultra-processed foods, which, in turn, was directly associated with poorer diet quality. Food insecurity mediated the indirect associations of socioeconomic status, availability of food at household, consumption of ultra-processed foods, and diet quality with HRQoL.

**Conclusion:**

The present findings elucidate the complex pathways between socioeconomic inequalities, food access and availability, diet quality, and children’s quality of life.

**Supplementary Information:**

The online version contains supplementary material available at 10.1007/s11136-025-04137-0.

## Abstract (plain language summary)

Children living in rural riverine communities face different health challenges because of social deprivation. Previous research showed that socioeconomic factors and food access are related to diet quality and quality of life. This study examined the possible interconnections between socioeconomic status, food availability, diet quality, food insecurity, and quality of life in this population. The results indicate that food insecurity was related to lower family socioeconomic status, less food at home, and poorer diet quality. Also, food insecurity was a meaningful pathway through which socioeconomic status, food availability, consumption of ultra-processed foods, and diet quality were associated with quality of life. These results highlight the intricate connections between socioeconomic factors, food access and food insecurity that affect children’s quality of life. Addressing food insecurity and improving diet quality could enhance children’s quality of life in these communities. These insights can help develop targeted interventions to improve children’s health and well-being.

**Supplementary Information:**

The online version contains supplementary material available at 10.1007/s11136-025-04137-0.

## Introduction

Social inequalities have been established to have a substantial influence on children’s health and quality of life [1, 2]. Health-related quality of life (HRQoL) is defined as the impact of health on a person’s capacity to live a fulfilling life, encompassing perceived physical functioning, mental and psychological well-being, social functioning, and the ability to perform everyday activities [3]. Contemporary theoretical frameworks on HRQoL acknowledge the importance of the biological, individual, and environmental factors [4]. The latter includes socioeconomic position and material circumstances that are particularly relevant for children’s HRQoL. Exposure to limited economic resources, such as low income, reduced consumption potential and poor household amenities impacts children’s health and HRQoL [1, 2]. However, the mechanisms by which socioeconomic inequalities influence children’s HRQoL are still poorly understood [4, 5].

Theories explaining socioeconomic health disparities often propose that lower socioeconomic status leads to insufficient material resources, placing people in the lowest end of the social gradient [5]. Therefore, material circumstances, including income, provide the necessary resources to nullify or reduce health risks in children that influence HRQoL [1, 6–8]. Children experiencing adverse circumstances are particularly more susceptible to poor nutrition-related health outcomes, since adverse childhood experiences are associated with unhealthy diet and food insecurity [9–11]. Moreover, the relationship between adverse childhood experiences, poor diet and food insecurity is stronger among socially vulnerable populations [9, 10]. Adverse childhood experiences were also related to poor diet quality and obesity [11]. Similarly, those living in vulnerable conditions and social deprivation are more likely to an unhealthy diet, undernutrition, chronic undernourishment, and food insecurity [2, 6, 7, 12]. Thus, socioeconomic inequalities can negatively affect children’s healthy diet and dietary patterns, their cognitive, behavioural, and socio-emotional development, and HRQoL [1, 8, 13, 14].

The role of socioeconomic status in infancy dietary patterns, nutritional status, and children’s development has been extensively reviewed [14]. In addition, a systematic review on the relationship between the diet quality, dietary behaviour and HRQoL in children and adolescents concluded that unhealthy dietary behaviour (e.g. irregular breakfast intake) or poor diet quality (e.g. high fat intake, fast food diet) were linked to reduced HRQoL [15]. Evidence suggests that undernutrition and food insecurity are critical issues affecting the health conditions and HRQoL of children [7, 13, 16–18]. Previous research carried out among urban low-income populations from developed countries demonstrated the relationship between dietary patterns, food insecurity, nutritional status and HRQoL [6, 8, 13, 18]. Fruits and raw vegetables intake and the mediterranean diet were associated with better HRQoL, whereas starchy foods and sweetened beverages were associated with poor HRQoL in children [8, 13, 19]. A previous study involving 10–14-year-old children in Tanzania showed that eating at eat three meals per day was associated with better HRQoL [7]. Moreover, Chilean children with healthy food habits and higher body mass index had higher odds of better HRQoL [13].

Even though there have been significant health improvements among children in recent decades, children from lower socio-economic backgrounds and those living in resource-limited communities continue to face vulnerabilities related to undernutrition and food insecurity [14, 20]. They represent a population group that is the least likely to follow the nutritional recommendations to promote health and prevent malnutrition due to a lack of, or irregular access to, a sufficient supply of nutritious food [14, 21]. Communities living in rural riverside areas of the Amazon region face significant social deprivation, characterised by limited access to essential services, such as healthcare, education, and sanitation. In addition, the prevalence of diseases and malnutrition is higher in these areas [22]. These communities often experience high levels of poverty and social exclusion, exacerbated by geographic isolation and inadequate infrastructure [23]. Challenges related to food insecurity and malnutrition in these communities are aggravated by geographic isolation and seasonal fluctuations in food availability [24]. The adverse living conditions of children from rural riverside communities in the Amazon predispose them to a higher risk of malnutrition, infectious diseases, and parasitic infections [25].

Unraveling the complex relationships between socioeconomic inequalities, diet quality, food access and availability, and children’s quality of life is crucial to understanding the pathways between social determinants and health outcomes. To the best of the authors’ knowledge, no prior studies have simultaneously assessed the relationships between social and living conditions, food availability, diet quality, nutritional status, food insecurity, and HRQoL in children from rural riverside areas of the Amazon region. We hypothesised that poor housing conditions, low socioeconomic status, low food availability at the household, greater consumption of ultra-processed foods and poor diet quality are associated with poor nutritional status, food insecurity and worse HRQoL among children. It was also conjectured that nutritional status and food insecurity mediate the relationship between housing conditions, socioeconomic status, availability of food at household, consumption of ultra-processed foods and diet quality with food insecurity and HRQoL.

## Methods

This study is reported according to AGReMA guideline for mediation analyses [27] and STROBE Checklist for cross-sectional studies [28].

### Study design and setting

A cross-sectional study was carried out in six communities located in the rural areas along the Solimões River and Lake Coari, in the city of Coari, Amazonas, Brazil. The city (population size = 70,616 inhabitants, 34.6% living in the rural areas) has low socioeconomic development, a poor standard of living, and substantial income inequality (HDI = 0.586; Gini Index = 0.623).

### Sampling procedures and study power

A representative sample of 5–10-year-old schoolchildren was selected from six primary public schools situated in the rural riverside communities. The present study included children between the ages of 5 and 10 enrolled in public schools, once they are particularly vulnerable to the negative impacts of inadequate nutrition and household food insecurity on their health, and cognitive and physical development [16]. Children in need of special educational services, those with disabilities, developmental disorders, or high abilities/giftedness were not included. A list of all students enrolled in the selected schools was obtained. Then, the parents of all potentially eligible students were invited to attend a meeting at the schools where the study’s aim and data collection procedures were explained. Those who agreed to participate were interviewed and their children were invited to complete the questionnaire and for clinical assessment.

The analysed sample size included 128 parent–child dyads, which granted a study power of 85%, considering structural equation modelling analysis with three latent variables and five observed variables, and a significance level of 5%, to detect statistically significant effects of at least 0.30 (medium effects) [28].

### Theoretical model

**Fig. 1:**
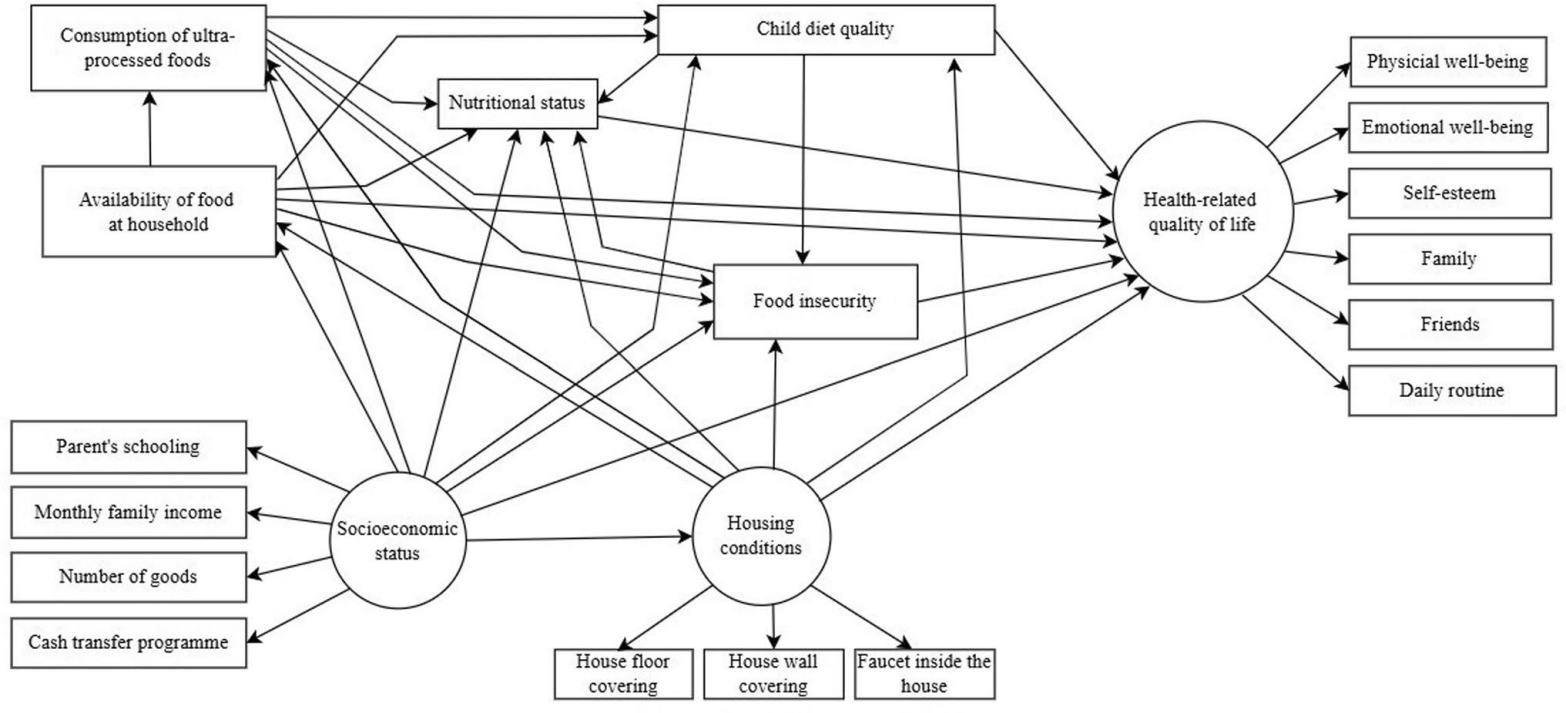
Full theoretical model on the relationships between socioeconomic status, household conditions, household food availability, consumption of ultra processed food, child’s diet quality, nutritional status, food insecurity and HRQoL in children living rural riverside areas in Amazonas, Brazil

The theoretical model (Fig. 1) hypothesised that greater socioeconomic status, adequate household conditions, higher household food availability, low consumption of ultra-processed foods, better diet quality would directly predict sound nutritional status, food security, and better HRQoL. In addition, better nutritional status and food security were hypothesised to predict better HRQoL. The theoretical model also hypothesised indirect effects of socioeconomic status, household conditions, household food availability, consumption of ultra-processed foods, diet quality on HRQoL via nutritional status and food security.

### Data collection

Schoolchildren aged between 5 and 10 years old and their parents were invited to obtain data on demographics (age and sex) and socioeconomic status (parental schooling, family income, number of goods household and registration in a cash transfer programme), housing conditions (house wall and floor covering and faucet inside the house), household food availability, consumption of ultra-processed foods, body mass index, child’s diet quality, food insecurity and HRQoL.

Individual interviews administered by trained researchers using standardised questionnaires were used to collect data in a private room at each school between March and June 2024. The participants’ parents were interviewed at the schools or their households to obtain information concerning socioeconomic status, housing conditions, household food availability and food insecurity. All questionnaires were administered using smartphones and the REDCap platform. Children’s weight was measured using a Premium portable digital scale, model AVA-350 Abs, Avanutri. Height was measured using a portable ultrasonic digital stadiometer, Avanutri, with an accuracy of 0.1 cm.

### Measures

Socioeconomic status was a latent variable using four indicators: parents’ schooling, monthly family income, number of goods, registration in a cash transfer programme. Parent’s schooling was assessed according to years of study using the response options: None = 1, 1–7 years = 2, 8–11 years = 3, and ≥ 12 years = 4. Monthly family income was registered in Brazilian reais according to the total earnings of the residents in the household. One US$ corresponded to 5.56 Brazilian reais during data collection. Number of goods in the household was evaluated based on an inventory of 21 durable items at home. Parents were asked whether the family was registered in the federal cash transfer programme (Bolsa Familia programme): 1 = no, 2 = yes.

Housing conditions was a latent variable measured by three indicators: house floor covering (1 = wood, 2 = ceramic or concrete/cement), house wall covering (1 = wood, 2 = brick), faucet inside the house (1 = no, 2 = yes).

Food availability at participants’ households in the last 30 days was assessed using the Household Food Availability Questionnaire. The absence (no = 0) or presence (yes = 1) of food and food products acquired through purchase, donation and/or own production was categorised into three groups according to the type of processing: fresh and/or minimally processed foods (16 items), culinary ingredients and processed foods (7 items), and ultra-processed foods (21 items). The items were summed to generate a score that could range from 0 to 44 [29].

Children's consumption of ultra-processed foods on the day prior to the interview was assessed using the Nova screening tool, comprising a list of 23 subgroups of ultra-processed foods that are presented to respondents with response options “no = 0” or “yes = 1”. The Nova Score was calculated based on the sum of the subgroups of ultra-processed foods, ranging from 0 to 23. The higher the Nova Score, the greater the consumption of ultra-processed foods [30].

The quality of the diet was assessed using the School Feeding Index Questionnaire (*Questionário Índice Alimentação do Escolar—Ales*), which is based on the Food Frequency Questionnaire assessing the usual frequency of consumption of 15 food items and one related to breakfast. The total score is obtained according to the frequency of positive and negative scores, following healthy eating recommendations for the Brazilian population. The frequency of individual values was added and distributed into tertiles, comprising three categories of food quality: poor quality (scores < 3), average quality (scores > 3 and < 6), and good quality (scores ≥ 6). Higher scores indicate better quality of diet [31, 32].

The Who Anthroplus software [33] was used to calculate the children’s body mass index (BMI) according to weight and height, expressed as a *z*-score, considering sex and age. Participant’s nutritional status was classified according to BMI for age following the World Health Organization cutt-offs: Underweight: < -2SD, Normal weight: ≥ -2SD to ≤  + 1SD, Overweight: >  + 1SD to ≤  + 2SD and Obesity/severe obesity: >  + 2SD [34, 35].

The 14-item Brazilian Food Insecurity Scale (BFIS) was used to assess household-level food insecurity [36, 37]. Parents were asked to answer food-related statements concerning the past three months: “no = 0”; “yes = 1”. Food insecurity status was coded into four categories based on the total number of affirmative responses to the BFIS as follows: food security = 0 points, mild food insecurity = 1–5 points, moderate food insecurity = 6–9 points, severe food insecurity = 10–14 points [37]. The assessment of food insecurity in Brazil based on BFIS is grounded in the principles outlined in General Comment No. 12 of the United Nations Committee on Economic, Social and Cultural Rights, which recognises the importance of upholding the right to safe, nutritious, affordable, and high-quality food, available in sufficient quantity and with permanent access to meet nutritional needs and support a healthy life [38].

Health-related quality of life (HRQoL) was assessed using the Brazilian versions of KINDL questionnaires for children aged 4–6 years (KiddyKINDL) and 7–13 years (KidKINDL) [39]. The KiddyKINDL and Kid-KINDL questionnaires comprise 12 and 24 items divided into six subscales: physical well-being, emotional well-being, self-esteem, family, friends, and daily routine (school). The total score of the KINDL is calculated by summing all items [39]. HRQoL was treated as a latent variable, with the scores of each dimension serving as indicators. Higher scores on KINDL subscales reflect better HRQoL.

### Statistical analysis

The descriptive analysis presented the distribution of variables through means and standard deviations for continuous variables, and proportions for categorical variables. Sensitivity analysis was conducted to compare sex and school year between study participants and non-participants using chi-square test and Fisher’s test. Confirmatory factor analysis (CFA) was employed to assess the measurement model encompassing three latent variables: socioeconomic status, housing conditions, and HRQoL. Structural equation modelling (SEM) was utilised to examine the direct and indirect relationships between observed and latent variables in accordance with the conceptual framework (Fig. 1), using SPSS AMOS 24.0 software.

The standardised total effects, comprising standardised direct effects (a direct path from one variable to another) and standardised indirect effects (a pathway mediated by other variables), were estimated. The 95% confidence intervals (95% CIs) were calculated using the maximum likelihood method via bias-corrected bootstrap to determine the presence of mediation by testing the statistical significance of the indirect effects, with 900 resamplings from the original dataset to derive less biased standard errors [40].

The adequacy of the measurement and structural models was evaluated based on the following fit indices and threshold values: χ^2^/df < 3.0, goodness of fit index (GFI) ≥ 0.90, comparative fit index (CFI) ≥ 0.90, standardised root mean square residual (SRMR) ≤ 0.08, and root mean square error of approximation (RMSEA) ≤ 0.06 [41]. Non-significant direct paths were removed from the full model to estimate a statistically parsimonious model.

### Ethical considerations

The present study was conducted in accordance with the Declaration of Helsinki, and the research protocol was approved by the Ethics Committee of the Federal University of Amazonas (Protocol no. 71554823.323.3.0000.5020) on the 23rd of August 2023. Initially, participants were thoroughly informed about the study's objectives and the data collection procedures. Parental and child informed consent were obtained before any data was collected. Participants were assured that their involvement was voluntary, they could withdraw at any point, and their responses would remain confidential.

## Results

Of the 255 schoolchildren deemed eligible from the selected schools, 128 were invited and accepted to participate (response rate = 100%). The remaining 127 (non-respondents = 49.8%) did not attend school or were not found in their homes during the period of data collection. Based on the information available in the school enrolment lists, a sensitivity analysis revealed no significant sex differences between the study sample and the schoolchildren who did not participate (*p* = 0.663). Participants were enrolled in earlier school years than non-participants, indicating that the former were likely younger than the latter (*p* = 0.024).

Family socioeconomic status, housing conditions, availability and consumption of food in the household, and children’s demographic characteristics and HRQoL are presented in Table 1. The mean monthly family income was BRL 1,624.2 and only 8.6% of the participants’ households were classified as with food security. The average age of the children was 7.9 years. Most children were males (53.1%) and had normal weight (75%) (Table 1). The Cronbach’s alpha coefficients of BFIS, KiddyKINDL and Kid-KINDL questionnaires were 0.843, 0.714 and 0.767, respectively..

**Table 1:** Socioeconomic status, housing conditions, household food availability and consumption, children’s demographics, diet quality, nutritional status and health-related quality of life

Variables	Total *N* = 128
*Socioeconomic status*	
Parents’ schooling	
None	7 (5.5)
1 to 7 years	48 (37.5)
8 to 11 years	72 (56.3)
≥ 12 years	1 (0.8)
Monthly family income (BRL), mean (± SD)	1,624.2 ± 794.3
Number of durable goods, mean (± SD)	8.4 ± 3.1
Registration in cash transfer programme	
No	16 (12.5)
Yes	112 (87.5)
*Housing conditions*	
House floor covering	
Wood	96 (75.0)
Ceramic or concrete/cement	32 (25.0)
House wall covering	
Wood	99 (77.3)
Brick	29 (22.7)
Faucet inside the house	
No	31 (24.2)
Yes	97 (75.8)
*Household food availability and consumption*	
Availability of food in the household, mean (± SD)	26.2 ± 5.5
Consumption of ultra-processed foods, mean (± SD)	4.8 ± 2.6
Food insecurity	
Food security	11 (8.6)
Mild food insecurity	70 (54.7)
Moderate food insecurity	34 (26.6)
Severe food insecurity	13 (10.2)
*Children characteristics*	
Age, mean (± SD)	7.9 ± 1.7
Sex	
Female	60 (46.9)
Male	68 (53.1)
Child’s diet quality	
Poor	83 (64.8)
Average	23 (18.0)
Good	22 (17.2)
Nutritional status	
Underweight	4 (3.1)
Normal weight	96 (75.0)
Overweight	24 (18.8)
Obesity/severe obesity	4 (3.1)
Health-related quality of life	
Total score, mean (± SD)	90.4 ± 10.6
Physical well-being, mean (± SD)	15.6 ± 2.9
Emotional well-being, mean (± SD)	15.7 ± 2.9
Self-esteem, mean (± SD)	13.7 ± 3.5
Family, mean (± SD)	16.2 ± 2.3
Friends, mean (± SD)	15.9 ± 2.3
Daily routine, mean (± SD)	13.3 ± 2.4

**Fig. 2:**
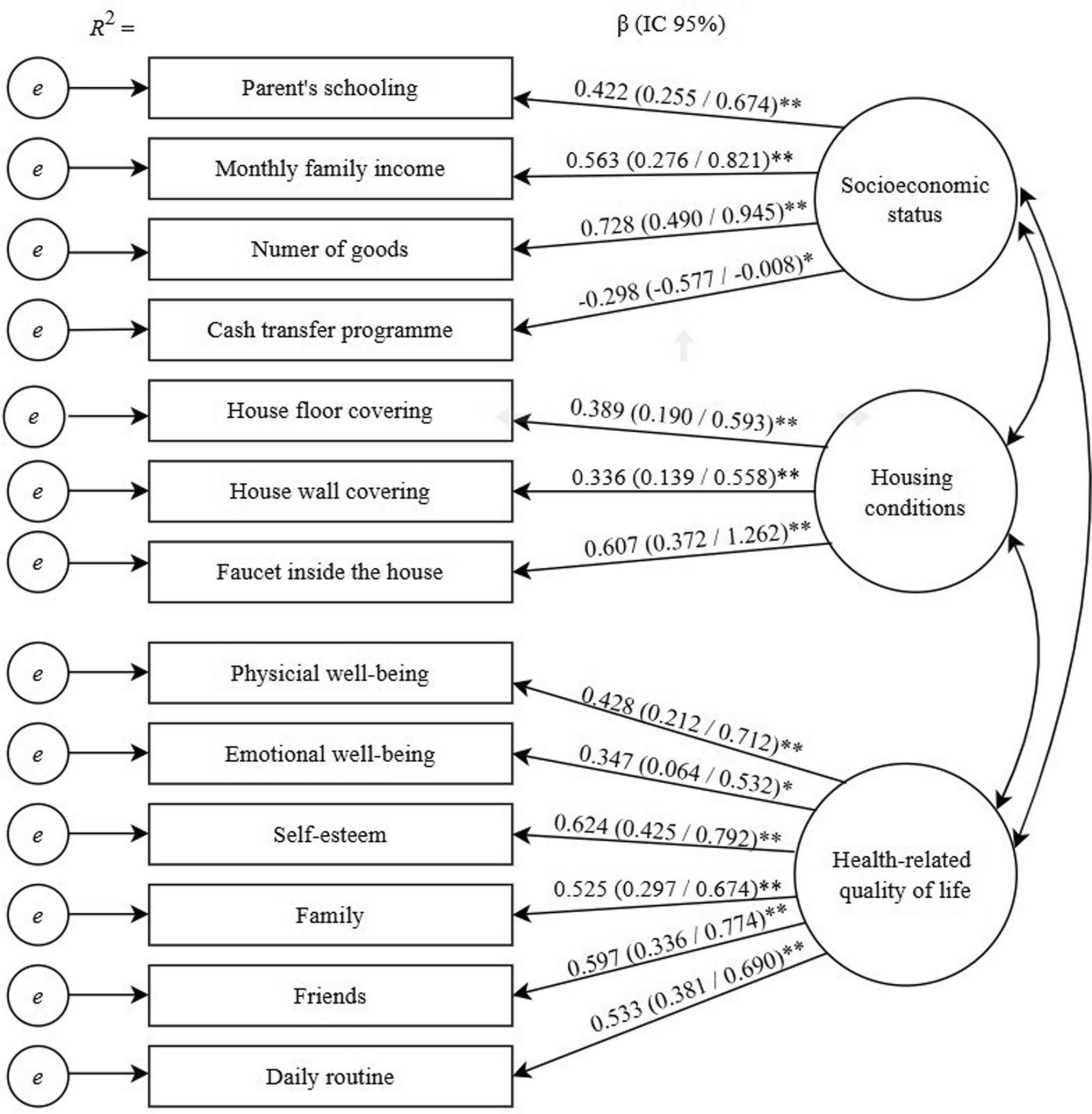
Confirmatory factor analysis of the 3-factor 6 items (measurement model) obtained through bootstrap item loadings (SE/BC 95% CI)

The full, measurement, and parsimonious models showed acceptable fit (Supplementary material 1). Confirmatory factor analysis (CFA) supported the three latent factors: socioeconomic status, housing conditions and HRQoL. Socioeconomic status comprised four indicators: parent’s schooling (β = 0.422), monthly family income (β = 0.563), number of goods (β = 0.728), and registration in a cash transfer programme (β = − 0.298). Housing conditions was composed of three indicators: house floor covering (β = 0.389), house wall covering (β = 0.336), and faucet inside the house (β = 0.607). HRQoL was composed of the KINDL dimensions: physical well-being (β = 0.428), emotional well-being (β = 0.347), self-esteem (β = 0.624), family (β = 525), friends (β = 597), and daily routine (β = 0.533) (Fig. 2).

**Fig. 3:**
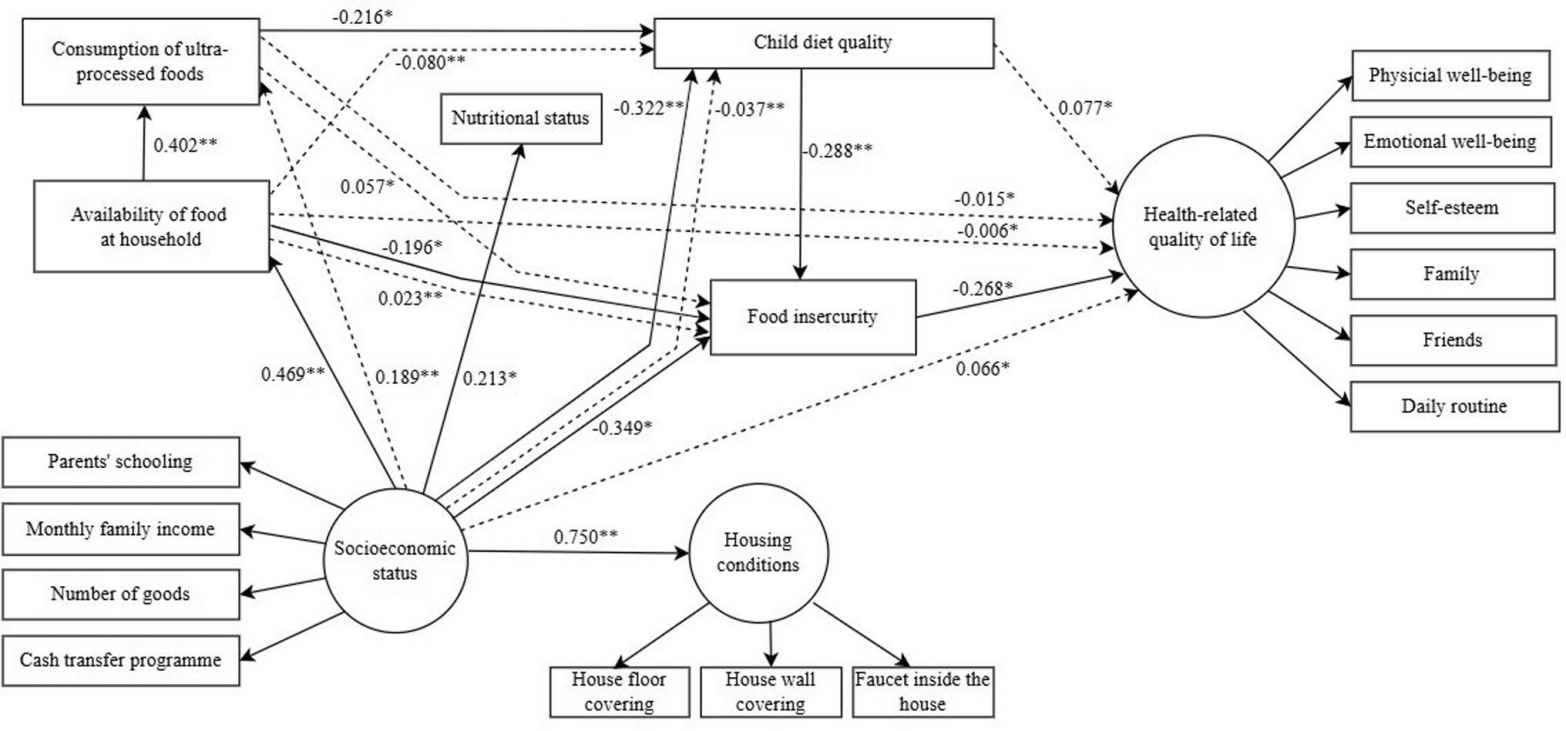
Parsimonious model of associations between socioeconomic status, household conditions, household food availability, consumption of ultra processed food, child’s diet quality, nutritional status, food insecurity and HRQoL. Direct effects are indicated by solid lines. Indirect effects are indicated by dashed lines. *P < 0.05, **P < 0.01

The standardised direct and indirect effects of the parsimonious are summarised in Fig. 3. Household food insecurity was directly predicted by worse socioeconomic status (β = − 0.349), lower availability of food at household food (β = − 0.196), and poorer child’s diet quality (β = − 0.288). Greater socioeconomic status level was directly associated with better housing conditions (β = 0.750), greater household food availability (β = 0.469), higher children’s BMI *z*-score (β = 0.213), and worse diet quality (β = − 0.322). Availability of food at the household was directly linked to higher consumption of ultra-processed foods (β = 0.402), which in turn was directly linked to worse diet quality (β = − 0.216). Household food insecurity was directly associated with poorer HRQoL (β = − 0.268).

Substantial indirect associations of socioeconomic status (β = 0.066), availability of food at household (β = − 0.006), consumption of ultra-processed foods (β = − 0.015), and diet quality (β = 0.077) with HRQoL mediated by food insecurity were identified. Moreover, socioeconomic status was indirectly linked to the consumption of ultra-processed foods (β = 0.189) and diet quality (β = − 0.037). Diet quality was indirectly linked to availability of food at household (β = − 0.080). Availability of food at household (β = 0.023) and consumption of ultra-processed foods (β = 0.057) were indirectly linked to food insecurity (Supplementary material 2).

## Discussion

The results of the study support the relationships between socioeconomic background, food availability and consumption, diet quality, food insecurity, and HRQoL in children aged between 5- and 10-years old living in rural riverside areas of the Amazon region. The present results suggest that children living in families experiencing food insecurity had poorer HRQoL. The higher the socioeconomic status, the higher the likelihood of higher availability of food and lower food insecurity in the household, and better HRQoL. In addition, several important indirect pathways by which contextual characteristics, including socioeconomic status, food availability and consumption of ultra-processed foods in the households, as well as child’s quality of diet can influence HRQoL were identified. Food insecurity proved to be the main mediator of these indirect associations.

Despite the present research including children and their parents living in rural riverside communities, the study’s findings are consistent with earlier research that has explored the relationship between adverse socioeconomic conditions, poor dietary quality and food insecurity in urban adult populations [42–45]. Previous studies have indicated that lack of money to purchase food [46, 47], household crowding [6] are significant risk factors for food insecurity. In addition, the relationship between economic deprivation and poor dietary quality has also been demonstrated [39, 43, 45]. The literature has documented that poor socioeconomic status and worse dietary behaviour are related to poor HRQoL in children [1, 2, 15]. However, evidence concerning the intricate relationships between social and economic conditions, pattern and quality of diet, food insecurity and HRQoL is scarce. Our study simultaneously assessed the role of socioeconomic status, availability and consumption of food, diet quality, food insecurity on HRQoL. Therefore, it was possible to reveal the potential pathways by which low socioeconomic background and poor dietary habits relate to HRQoL. Since food insecurity is disproportionately concentrated in poor rural families [20], the present findings provide a sound understanding of the mediation effect of food insecurity on the association between poor socioeconomic status, unhealthy dietary habits and HRQoL in children living in deprived social conditions.

The hypothesis of the negative effects of low food availability, higher consumption of ultra-processed foods and poor diet quality on children’s nutritional status was not confirmed. Similarly, the mediation effect of nutritional status on the association of socioeconomic status, food availability, consumption of ultra-processed foods and diet quality with HRQoL was not observed. The above-mentioned results might have occurred due to the predominance of children with normal weight (75% of the sample) and the very small number of underweight children (3%). Although this finding seems unexpected for a group of socially deprived children, the occurrence of children’s underweight is uncommon in the North region of Brazil due to the available fishing in the rivers of the region [9, 11]. In addition, the discrepancy between the preponderance of children with normal weight and those living in households with food insecurity (91%) may result from the multidimensional concept of food security adopted in Brazil encompassing food quantity, quality, adequacy and sustainability [38]. Mild and moderate food insecurity do not imply a total lack of food, but rather inconsistent and unreliable access to sufficient, quality food [48, 49]. Individuals may therefore be eutrophic or even overweight. When income is low and future food availability uncertain, dietary choices are driven by affordability and satiety. This often results in diet dominated by low-cost, high-dense foods, minimal intake of unprocessed items (in natura) foods, and limited variety (monotony) [48, 49]. Consequently, children may consume enough calories to maintain (eutrophy) or gain weight (overweight), yet still suffer from micronutrient deficiencies.

Even though food insecurity and poor diet quality were very common in this sample, the high consumption of ultra-processed foods may explain the reasonable number of children who apparently had their nutritional needs met and were eutrophic. On the other hand, a significant proportion of children was overweight (21.9%), indicating that, similar to other regions of high social vulnerability, the nutritional transition is underway in riverine communities in the Amazon [50].

The current study benefits from the strengths of using structural equation modelling statistical method, which allows for the concomitant analysis of both direct and indirect complex relationships between variables and the assessment of mediation effects within a conceptual framework [51]. In this research, socioeconomic status, housing conditions and HRQoL were examined as latent variables to represent multidimensional constructs. The study addresses the common limitation of assessing social status through a single variable by using parents’ schooling, monthly family income, and number of goods as indicators of socioeconomic status. Similarly, three relevant indicators pertaining to the physical conditions of the house were used to assess housing conditions. Using the scores of subscales’ dimensions of KINDL to represent HRQoL as a latent variable yielded more reliable data and minimised measurement errors.

Our findings should be interpreted in light of their limitations. The participants comprised a specific population of children from rural riverside areas. Therefore, our results should not be generalised for urban populations and other age groups. Although the sample size was sufficient to identify a structural equation model with robust power, and identify several significant direct and indirect relationships, the number of participants was insufficient to detect associations with small effect sizes. The cross-sectional design constrains the interpretation of causal processes underlying the associations proposed in the theoretical model. Moreover, the direct association between lower availability of food in the household and food insecurity could be due to reverse causation. A previous review suggested that food insecurity itself leads to lower food availability [44]. Thus, the direction of the hypothesised relationship might be the opposite of what was initially assumed. Recent evidence suggests that adverse childhood experiences, including household dysfunction, are associated with food insecurity, quality of diet and nutritional status [9–11]. Future research should investigate the mediating effect of nutritional status, diet and food insecurity on the relationship between adverse childhood experiences and HRQoL. Finally, psychological factors were identified as potential mechanisms underlying the association between household food insecurity and children’s quality of life [52]. In this study, other potentially relevant predictors and mediators for HRQoL in children and adolescents, such as mental health problems and psychosocial factors, identified in previous research should be considered in upcoming studies [53, 54].

This work identifies potential implications for local and national social, educational, and agricultural policy agendas. Inter-sectoral policies to reduce social inequalities between urban better-off groups and rural riverside people, and to decrease poverty in the latter population are urgently needed. They may include promoting adult schooling through adapting and expanding existing governmental educational programmes, such as the Youth and Adult Education (EJA) [55], as well as initiatives aimed at employment and income generation. Such approaches can potentially enhance dietary habits, reduce food insecurity and improve children’s HRQoL. Riverside dwellers living in rural areas of Amazonas have been facing a dietary transition since the diet of traditional foods has been gradually decreasing and being replaced by industrialised and processed foods due to urbanization, which impacts riverside lifestyles [56]. Therefore, educational actions aimed at valuing traditional local foods and eating practices of riverside communities can help preserve local culture and ensure that children have access to a healthy and diverse diet [56]. In addition, governmental initiatives to promote family farms and community-oriented gardens that consider the local social and cultural aspects of rural riverside populations should be developed and implemented. Agricultural public policies aimed at sustainable practices supporting local food production would likely reduce the dependency on external food sources and mitigate the risks associated with food supply disruptions and food insecurity [57, 58]. Community gardens can provide access to fresh and nutritious food that can lead to better dietary habits, a healthy lifestyle, and overall health outcomes [57, 58]. Ultimately, family farms and community gardens contribute to local economies by creating job opportunities [59].

## Conclusion

This is the first study to provide evidence of the significance of socioeconomic status, availability of food in the household, consumption of ultra-processed foods and quality of diet on HRQoL as well as the mediating effect of food insecurity on the relationship between socioeconomic status, housing, food availability and consumption, and HRQoL among children from rural riverside communities of the Amazon region. Our findings underscore the need for public policies focusing on social and agricultural development, particularly those that address the unique needs of rural and underserved communities, to enhance their overall quality of life.

## Supplementary Information

Below is the link to the electronic supplementary material.

Supplementary Material 1

Supplementary Material 2
