# Supplementary material for: Associations between socioeconomic status, dietary habits and health-related quality of life among children in rural riverside communities: the mediation role of food insecurity
Source: Qual Life Res. 2026 Jan 9;35(2):41. doi: 10.1007/s11136-025-04137-0 (PMC12789232; doi:10.1007/s11136-025-04137-0)
Supplement: Supplementary file 1 — Supplementary Material 1 [file 11136_2025_4137_MOESM1_ESM.docx]

**Title:** Associations between socioeconomic status, dietary habits and health-related quality of life among children in rural riverside communities: the mediation role of food insecurity

**Journal:** Quality of life Research

**Authors:** Luziane de Lima Pereira, Fernando José Herkrath, Jordana Herzog Siqueira, Maria do Carmo Leal, Fabíola Macedo de Abreu, Amanda Forster Lopes, Mario Vianna Vettore*

***Correspondig author**

Mario Vianna Vettore

Department of Dentistry and Oral Health, Aarhus University, Aarhus, Denmark.

Email address: m.vettore@dent.au.dk

**Supplementary material 1. Fit indices for the confirmatory factor analysis of full, measurement**

**and parsimonious models.**

| **Model** | **χ^2^/**df | **GFI** | **CFI** | **SRMR** | **RMSEA** |
| --- | --- | --- | --- | --- | --- |
| Full | 1.405 | 0.89 | 0.91 | 0.08 | 0.06 |
| Measurement model | 1.263 | 0.92 | 0.96 | 0.07 | 0.05 |
| Pasimonious | 1.225 | 0.90 | 0.95 | 0.08 | 0.04 |
