# Supplementary material for: Associations between socioeconomic status, dietary habits and health-related quality of life among children in rural riverside communities: the mediation role of food insecurity
Source: Qual Life Res. 2026 Jan 9;35(2):41. doi: 10.1007/s11136-025-04137-0 (PMC12789232; doi:10.1007/s11136-025-04137-0)
Supplement: Supplementary file 2 — Supplementary Material 2 [file 11136_2025_4137_MOESM2_ESM.docx]

**Title:** Associations between socioeconomic status, dietary habits and health-related quality of life among children in rural riverside communities: the mediation role of food insecurity

**Journal:** Quality of life Research

**Authors:** Luziane de Lima Pereira, Fernando José Herkrath, Jordana Herzog Siqueira, Maria do Carmo Leal, Fabíola Macedo de Abreu, Amanda Forster Lopes, Mario Vianna Vettore*

***Correspondig author**

Mario Vianna Vettore

Department of Dentistry and Oral Health, Aarhus University, Aarhus, Denmark.

Email address: m.vettore@dent.au.dk

Supplementary material 2. Standardised direct and indirect effects of the parsimonious model.

| **Direct effects** | **β** | **IC 95%** | ***P*** |
| --- | --- | --- | --- |
| Socioeconomic status→ Availability of food at household food | 0.469 | 0.294 / 0.654 | 0.002 |
| Socioeconomic status→ BMI *z*-score | 0.213 | 0.011 / 0.383 | 0.040 |
| Socioeconomic status→ Child’s diet quality | -0.322 | -0.487 / -0.149 | 0.004 |
| Socioeconomic status→ Food insecurity | -0.349 | -0.605 / -0.081 | 0.010 |
| Socioeconomic status→ Housing conditions | 0.750 | 0.443 / 1.148 | 0.002 |
| Availability of food at household food → Consumption of ultra-processed foods | 0.402 | 0.276 / 0.527 | 0.001 |
| Child’s diet quality → Food insecurity | -0.288 | -0.454 / -0.096 | 0.009 |
| Food insecurity → HRQoL | -0.268 | -0.487 / -0.048 | 0.023 |
| **Indirect effects** |  |  |  |
| Socioeconomic status → Consumption of ultra-processed foods | 0.189 | 0.101 / 0.319 | 0.001 |
| Socioeconomic status → Child’s diet quality | -0.037 | -0.081 / -0.011 | 0.004 |
| Socioeconomic status → HRQoL | 0.066 | 0.001 / 0.179 | 0.012 |
| Availability of food at household food → Child’s diet quality | -0.080 | -0.153 / -0.018 | 0.008 |
| Availability of food at household food → Food insecurity | 0.023 | 0.006 / 0.051 | 0.007 |
| Availability of food at household food → HRQoL | -0.006 | -0.019 / -0.001 | 0.016 |
| Consumption of ultra-processed foods → Food insecurity | 0.057 | 0.012 / 0.114 | 0.010 |
| Consumption of ultra-processed foods → HRQoL | -0.015 | -0.042 / -0.002 | 0.015 |
| Child’s diet quality → HRQoL | 0.077 | 0.015 / 0.181 | 0.016 |
